# Fatal Cerebral Air Embolism: A Case Series and Literature Review

**DOI:** 10.1155/2016/3425321

**Published:** 2016-08-21

**Authors:** Rashmi Mishra, Pavithra Reddy, Misbahuddin Khaja

**Affiliations:** ^1^Division of Pulmonary and Critical Care Medicine, Bronx Lebanon Hospital Center Affiliated to Icahn School of Medicine at Mount Sinai, 1650 Grand Concourse, Bronx, NY 10457, USA; ^2^Department of Medicine, Bronx Lebanon Hospital Center Affiliated to Icahn School of Medicine at Mount Sinai, 1650 Grand Concourse, Bronx, NY 10457, USA

## Abstract

Cerebral air embolism (CAE) is an infrequently reported complication of routine medical procedures. We present two cases of CAE. The first patient was a 55-year-old male presenting with vomiting and loss of consciousness one day after his hemodialysis session. Physical exam was significant for hypotension and hypoxia with no focal neurologic deficits. Computed tomography (CT) scan of head showed gas in cerebral venous circulation. The patient did not undergo any procedures prior to presentation, and his last hemodialysis session was uneventful. Retrograde rise of venous air to the cerebral circulation was the likely mechanism for venous CAE. The second patient was a 46-year-old female presenting with fever, shortness of breath, and hematemesis. She was febrile, tachypneic, and tachycardic and required intubation and mechanical ventilation. An orogastric tube inserted drained 2500 mL of bright red blood. Flexible laryngoscopy and esophagogastroduodenoscopy were performed. She also underwent central venous catheter placement. CT scan of head performed the next day due to absent brain stem reflexes revealed intravascular air within cerebral arteries. A transthoracic echocardiogram with bubble study ruled out patent foramen ovale. The patient had a paradoxical CAE in the absence of a patent foramen ovale.

## 1. Introduction

The introduction of air into cerebral arterial or venous circulation called cerebral air embolism (CAE) can lead to severe neurologic deficits and possible death. It may be iatrogenic secondary to central venous catheter (CVC) placement or removal, endoscopy, hysteroscopy, laparoscopy, and defibrillator placement [1–4]. Cases of CAE have been reported after hemodialysis despite the safeguards associated with modern hemodialysis machines [5]. We report two cases of CAE. The first was a case of CAE one day after an uneventful hemodialysis session, and the second was a case of CAE occurring after the patient underwent esophagogastroduodenoscopy (EGD), laryngoscopy, and CVC placement.

## 2. Case Presentation

**Figure 1:**
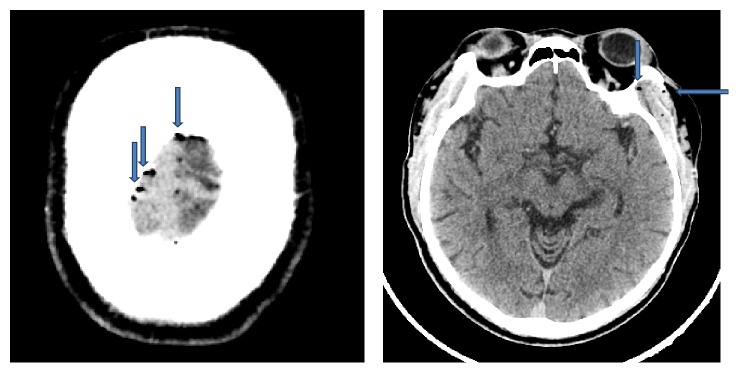
Case 1 CT scan of head: axial cuts showing foci of gas in cerebral vein and left temporal muscle (blue arrows).

*Case 1*. The patient was a 55-year-old male who presented to the hospital with multiple episodes of vomiting, loss of consciousness, and repeated falls over the last day. He denied any chest pain, palpitations, or injury to his head. His medical history was significant for hypertension, diabetes mellitus, and end-stage renal disease. He received hemodialysis through his left forearm arteriovenous fistula one day prior to presentation. On presentation, the patient was hypotensive, with blood pressure of 90/50 mm Hg, was hypoxic, with an oxygen saturation of 88% of room air, had a heart rate of 79 beats per minute, and was afebrile. On physical exam, he was alert and oriented, with negative respiratory and cardiovascular exams. Neurological exam did not show any focal neurologic deficits. Laboratory data were significant for thrombocytopenia with a platelet count of 122 × 10^3^/*μ*L and a hemoglobin level of 17.3 g/dL. He underwent a CT scan of his head, which showed foci of gas in the cavernous sinus, anterior sagittal sinus, left side of the face, and left temporalis muscle (Figure 1). He had not undergone any other procedures before presentation, and, by history, his hemodialysis had been uneventful. The patient's condition deteriorated rapidly; he became comatose and had a cardiac arrest. He was successfully resuscitated within five minutes. After resuscitation, the patient developed shock requiring vasopressor support. He was started on intravenous piperacillin-tazobactam and vancomycin for possible sepsis. In the presence of high pulmonary pressures and without any evidence of paradoxical air embolism, a bubble study to look for a patent foramen ovale was not performed. Adequate oxygenation was maintained throughout his hospital stay. He was not a candidate for transfer to another center for possible hyperbaric oxygen therapy due to his tenuous hemodynamic status. The patient's further course was complicated by hematemesis due to a Mallory-Weiss tear diagnosed by endoscopy. Sepsis work-up was negative. As his prognosis was poor, he was transferred to hospice care where he expired.

**Figure 2:**
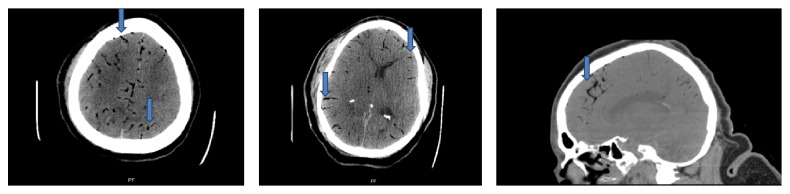
Case 2 CT scan of head: axial and sagittal cuts showing foci of gas in bilateral cerebral arteries (blue arrows).

*Case 2*. The patient was a 46-year-old female who presented to the emergency room with a one-day history of fever, shortness of breath, hematemesis, and abdominal pain. Her past medical history included hypertension, chronic systolic congestive heart failure, atrial fibrillation with implantable cardioverter-defibrillator (ICD) placement and accessory pathway ablation two weeks prior to presentation, and coronary artery disease. Vital signs included fever, with a temperature of 39.3°C, tachypnea, and tachycardia. Worsening tachypnea and tachycardia necessitated endotracheal intubation for acute respiratory failure. An orogastric tube was placed and drained 2500 mL of bright red blood. Flexible laryngoscopy and EGD did not reveal an upper airway or upper gastrointestinal (GI) source of bleeding. The patient eventually required placement of a CVC for administration of fluids, blood products, and vasopressors. She was admitted to the medical intensive care unit with a working diagnosis of hemorrhagic shock secondary to an upper GI bleed and sepsis secondary to healthcare-associated pneumonia. She was started on esomeprazole and octreotide drips and empiric antibiotics. On the second day after admission, neurologic examination without sedation revealed absent brain stem reflexes. CT scan of the head performed revealed extensive intravascular air within numerous cerebral arteries bilaterally, diffuse cerebral edema, and tonsillar herniation (Figure 2). A transthoracic echocardiogram with bubble study ruled out a patent foramen ovale. Bedside apnea testing confirmed brain death. Autopsy revealed severe dilated cardiomyopathy with no obvious cause of CAE.

## 3. Discussion

Cerebral air embolism can be a potentially lethal complication. It can occur iatrogenically, secondary to CVC placement or removal, endoscopy, hysteroscopy, laparoscopy, or defibrillator placement, among other reported causes [1–4]. It has been reported in relation to hemodialysis by Hysell [5]. In the past, CAE with a demonstrable air column in the hemodialysis circuit has been reported [6]. Use of a hemodialysis catheter outside of hemodialysis and declotting of hemodialysis access have also been reported as causes of CAE [7, 8]. With the advent of modern hemodialysis, there are safety measures in hemodialysis machines to protect against CAE, but it may still occur. Microbubbles have been noted in hemodialysis circuits. They may originate in arterial luer lock connector at negative pressure or from remnant bubbles due to insufficient priming, but in some cases, the source remains unknown. These may not be picked up as air by the dialysis circuit. Blood flow rate and negative arterial pressure correlate with the microbubble rate as per a recent observational study [9]. The microbubbles get lodged in the capillaries and cause tissue ischemia, inflammatory response, and complement activation. Furthermore, obstruction of microcirculation and tissue damage occur due to platelet aggregation and clot formation [10].

Iatrogenic CAE related to CVC has been widely reported. In a systematic review of iatrogenic CAE related to CVC, the most common locations for emboli, in decreasing order of frequency, are in the subarachnoid space, cerebral parenchyma, and venous sinus. The most common neurologic symptoms reported were focal neurologic deficits, coma, seizures, encephalopathy, and headache. The time to symptom onset has been reported to be very acute, with the median time to onset reported by the review being one minute [11]. Iatrogenic CAE has been reported with CVC placement, manipulation, and removal [12–15]. The risk of air embolism is higher in conditions with decreased central venous pressure like hypovolemia, deep inspiration, or patient in upright position [15].

CAE secondary to endoscopy and other GI procedures, such as endoscopic retrograde cholangiopancreatography, have also been previously reported. It is thought that air insufflation required for EGD creates pressure gradient favoring passage of air into the vasculature [16, 17].

The mechanism for entrance of air into the cerebral venous circulation is thought to be through retrograde rise. Air, once it enters the venous circulation, can rise in an upright patient to the cerebral venous circulation at a speed greater than the venous blood flow due to its low specific gravity [18]. The rise of air depends further on factors like bubble size, central vein diameter, and cardiac output [19]. Loss of consciousness, seizures, and possible death can occur as a result of the CAE [20]. Retrograde cerebral venous air embolism to the sagittal sinus and cortical veins has been reported in association with CVC disconnection and the use of a CVC in a mobile patient [12, 13]. If the patient is recumbent, air can enter the heart and pass into the ventricle and lungs, causing dyspnea, cough, chest tightness, and arrhythmias [20].

Arterial CAE, on the other hand, may occur if air passes from the venous into the arterial circulation. This may occur due to the presence of a right-to-left shunt, such as a patent foramen ovale. In the absence of an obvious right-to-left shunt, pulmonary arteriovenous malformations may cause paradoxical CAE. Inability of the pulmonary vasculature to filter out emboli due to the presence of a large volume venous embolism or the use of anesthetic agents may also cause paradoxical CAE [4, 14]. Animal studies show that lung, which can act as a physiologic filter, can become overwhelmed above 0.3 mL/kg/min, but exact filtering capacity of human lung is unknown [21]. Pathologic dilation of pulmonary vessels or pulmonary AV malformations may occur in chronic liver disease or in patients with hereditary hemorrhagic telangiectasia [22]. Our patient with arterial CAE did not have history suggestive of either. Intrapulmonary arteriovenous malformations closed at rest, but which can open during exercise or other hyperdynamic conditions, are present in more than 90% of humans. These intrapulmonary anastomoses can be affected by oxygen tension and by body positioning. Once these pathways open, these pathways can be closed by FiO2 = 1.0 during submaximal-through-maximal exercise in most healthy humans [23].

The treatment of venous CAE consists of volume resuscitation to increase venous pressure in order to prevent continued entry of air into the venous circulation. Adequate oxygenation should be attained, with an increase in the fraction of inspired oxygen (FiO2) of inspired gas. Increasing the FiO2 also helps to decrease the size of the air embolus by increasing the gradient for nitrogen. Hyperbaric oxygen therapy is not the first line of therapy for venous CAE. If there is evidence of neurologic deficits, it may be considered [4].

The treatment of arterial CAE consists of supportive treatment. Endotracheal intubation, ventilation, and administration of oxygen to maintain and improve oxygenation and decrease the size of gas bubbles are recommended. Suppression of seizures with benzodiazepines or barbiturates should be performed. Hyperbaric oxygen therapy helps by raising ambient pressure around the gas bubble and increasing the gradient for nitrogen out of the bubble and for oxygen into the bubble. Hemoconcentration may occur with gas embolism, which can cause increased viscosity and compromise microcirculation. Normovolemia should be attained with infusion of colloids. Use of anticoagulation and corticosteroids is not recommended [4].

Our first patient presented the next day after undergoing hemodialysis with symptoms of syncope, recurrent falls, and vomiting. Initially, he was alert and able to provide history. In the venous CAE case reported by Brouns et al., the patient also had syncope one day prior to the development of coma [12]. Other cases have also reported delayed symptoms after retrograde venous CAE [24]. The arterial needle, prepump arterial tubing segment, and inadvertently opened end of a CVC are common sites of air entry [20]. In our patient, the mechanism of air entry into the venous circulation is unclear. Microbubbles during hemodialysis may be a possible explanation. Once air entered the venous circulation, retrograde cerebral venous air embolism seems to be the mechanism of the CAE.

Our second patient underwent multiple procedures including laryngoscopy, endoscopy, and placement of a CVC, any of which could have caused the CAE. Due to the absence of a patent foramen ovale, the mechanism for the paradoxical CAE was likely the inability of the pulmonary circulation to filter out the air embolus from the venous circulation.

## 4. Conclusion

Sudden cardiac and cerebral dysfunction in mechanically ventilated patients with risk factors should suggest CAE, especially in the setting of invasive endoscopic procedures and/or CVC placement, which are common in any intensive care unit. Also, CAE is uncommon in the era of modern hemodialysis, but this may still occur, as is evident from the first case. The first case is also interesting in that the patient showed delayed symptoms after his hemodialysis session. CAE should be considered in the differential diagnosis of hemodialysis patients presenting with neurologic symptoms at any time. These cases bring to light the need for awareness among physicians regarding the possibility of this complication following routine medical procedures.

## Abbreviations

CAE: Cerebral air embolism
CVC: Central venous catheter
EGD: Esophagogastroduodenoscopy
CT: Computed tomography.
